# Issues of interspecific hybridization between Solanum lycopersicum L. and Solanum sisymbriifolium Lam.

**DOI:** 10.18699/vjgb-26-59

**Published:** 2026-07

**Authors:** A.V. Vishnyakova, A.P. Fedotov, E.V. Osminina, A.Z. Martirosyan, A.D. Kobyashova, A.A. Alexandrova, A.A. Mironov, S.G. Monakhos

**Affiliations:** Russian State Agrarian University – Moscow Timiryazev Agricultural Academy, Moscow, Russia; Lomonosov Moscow State University, Moscow, Russia; Skolkovo Institute of Science and Technology, Moscow, Russia; Russian State Agrarian University – Moscow Timiryazev Agricultural Academy, Moscow, Russia; Russian State Agrarian University – Moscow Timiryazev Agricultural Academy, Moscow, Russia; Russian State Agrarian University – Moscow Timiryazev Agricultural Academy, Moscow, Russia; Russian State Agrarian University – Moscow Timiryazev Agricultural Academy, Moscow, Russia; Russian State Agrarian University – Moscow Timiryazev Agricultural Academy, Moscow, Russia

**Keywords:** tomato, sticky nightshade, Solanum lycopersicum, Solanum sisymbriifolium, interspecific hybridization, embryo rescue, microsporogenesis, embryology, mixoploidy, томат, паслен гулявниколистный, Solanum lycopersicum, Solanum sisymbriifolium, межвидовая гибридизация, эмбриокультура, микроспорогенез, эмбриология, миксоплоидия

## Abstract

Interspecific hybridization plays a crucial role in tomato (Solanum lycopersicum L.) breeding for introducing beneficial traits from wild relatives, such as resistance to biotic and abiotic stress. Sticky nightshade (Solanum sisymbriifolium Lam.) is a promising source for the introgression of desirable traits. Reports on hybridization between S. lycopersicum and S. sisymbriifolium remain contradictory, differently describing the progeny as doubled haploids or interspecific hybrids. In the present study, we clarify the nature of the progeny derived from hybridization between S. lycopersicum and S. sisymbriifolium by analyzing the early stages of ovule development and characterizing the morphological and cytogenetic features of the resulting plants. Interspecific hybridization between S. lycopersicum and S. sisymbriifolium encounters postzygotic reproductive barriers. Only a small proportion of developing ovules in male-sterile tomato lines, whether self-pollinated or pollinated with S. sisymbriifolium followed the normal developmental pathway. In most cases, either ovule development was arrested, or parthenocarpic seed-like bodies formed instead of normal seeds. Embryo rescue enabled the recovery of 12 plants resulting from interspecific hybridization of S. lycopersicum and S. sisymbriifolium. Morphologically, the plants closely resembled the S. lycopersicum parent, although they displayed several traits distinctive from those of the parental tomato lines. Notably, yellow-orange mature fruits developed in progeny from the cross of green-fruited S. lycopersicum and red-fruited S. sisymbriifolium. Analysis of chromosome numbers in root meristems revealed mixoploidy (2n = 16–26), and meiotic analysis during microsporogenesis showed multiple aberrations in meiosis. Thus, comprehensive embryological, morphological, and cytogenetic analyses provide evidence for the hybrid origin of the obtained plants. This confirms the possibility of overcoming postzygotic barriers between these species and opens avenues for the utilization of S. sisymbriifolium in tomato breeding programs.

## Abstract (ru)

Межвидовая гибридизация играет большую роль в селекции томата (Solanum lycopersicum L.), позволяет обогащать генофонд новыми признаками и создавать сорта и гибриды с устойчивостью к биотическим и абиотическим факторам. Перспективным источником для интрогрессии ценных признаков может быть паслен гулявниколистный (Solanum sisymbriifolium Lam.). Сведения о результатах гибридизации между S. lycopersicum и S. sisymbriifolium противоречивы: одни исследователи описывают полученные растения как удвоенные гаплоиды, другие – как типичные межвидовые гибриды. В настоящей работе мы уточнили природу потомства от гибридизации S. lycopersicum и S. sisymbriifolium путем анализа начальных стадий развития семязачатков и описания морфологических и цитогенетических особенностей полученных растений. Получение межвидовых гибридов между S. lycopersicum и S. sisymbriifolium сопряжено с постзиготическими барьерами нескрещиваемости. Сравнительный анализ развивающихся семязачатков от самоопыления линий томата с функциональной мужской стерильностью и от их опыления S. sisymbriifolium выявил, что лишь часть семязачатков развивается по типичному пути, в то время как большинство либо останавливается в развитии, либо формирует партенокарпические структуры с псевдоэмбриональной тканью. С использованием эмбриокультуры нами было получено 12 растений от гибридизации S. lycopersicum и S. sisymbriifolium. По морфологическим признакам все растения были близки S. lycopersicum, однако отмечено появление признаков, не характерных для родительских линий томата, например желто-оранжевой окраски зрелых плодов у растений, полученных при скрещивании линии зеленоплодных томатов с красноплодным пасленом гулявниколистным. При анализе числа хромосом в меристемах корня наблюдали миксоплоидию (2n = 16–26), а при микроспорогенезе регистрировали множественные нарушения мейоза. Таким образом, комплексный эмбриологический, морфологический и цитогенетический анализ предоставляет доказательства гибридного происхождения полученных растений, что подтверждает возможность преодоления постзиготических барьеров между данными видами и открывает путь к использованию S. sisymbriifolium в селекции томата.

## Introduction

Tomato (Solanum lycopersicum L.) is one of the world’s most economically important vegetable crops. However, its productivity is significantly limited by its susceptibility to biotic and abiotic stresses (Sadashiva et al., 2017). Thus, breeding strategies that develop genotypes with genetic resistance are crucial (Abbas et al., 2024). One of the key approaches to solving this problem is interspecific hybridization, which enables the introduction of valuable alleles from related wild species into the cultivated tomato genome (Ajaharuddin et al., 2024).

The introgression of disease resistance genes from other species into tomatoes has been employed for long and is quite successful (Rubio et al., 2016; Yerasu et al., 2023). Currently, most of the sources of resistance used in tomato breeding are wild species of the Lycopersicon section. Species from other sections of the Solanum genus are in lesser use (Foolad, 2007), as their gene pool availability is restricted by incompatibility barriers (Abbas et al., 2024).

Sticky nightshade (Solanum sisymbriifolium Lam.), a distant relative of the tomato, is regarded by some researchers as a promising source of disease resistance. It has been found to be resistant to bacterial wilt (Collonnier et al., 2003) and verticillium wilt (Piosik et al., 2019). According to W.G. Flier et al. (2003), S. sisymbriifolium exhibits hypersensitivity when inoculated with several European isolates of Phytophthora infestans, a potato pathogen. In field tests in central Russia, S. sisymbriifolium demonstrated high resistance to tomato late blight, according to our observations. Additionally, researchers have reported resistance to carmine spider mites (Piosik et al., 2019) and nematodes (Hajihassani et al., 2020). S. sisymbriifolium has a high adaptive potential and is currently widespread in many countries (Biswas et al., 2023).

Hybridization between distantly related species, such as tomato and sticky nightshade, encounters serious post-zygotic barriers. However, it has been demonstrated that sticky nightshade pollen tubes successfully grow within tomato pistils and germinate in ovules (Bal, Abak, 2003; Piosik et al., 2019). Interspecific hybridization between S. lycopersicum and S. sisymbriifolium has been attempted by several research teams. However, the results were inconsistent: D. Chambonnet (1996) and U. Bal and K. Abak (2003) obtained plants phenotypically similar to tomatoes and considered them to be doubled haploids. In contrast, Ł. Piosik et al. (2019) produced interspecific hybrids that resembled nightshades. Thus, the nature of plants obtained through interspecific hybridization, as well as their morphological and cytological characteristics, remains obscure

The goal of this study was to produce plants resulting from the cross between S. lycopersicum and S. sisymbriifolium and to perform a comprehensive analysis of their characteristics using comparative embryology, cytogenetics, and morphological trait analysis.

## Materials and methods

Plant material and hybridization. The tomato samples used for hybridization were obtained from the Timofeev Breeding Station, Moscow. These samples include beef tomato lines Roz Son and Roz st9 and cherry tomato lines st8, st6, st6(lik), and st4. Lines Roz Son, st8, st6, st6(lik), and st4 are generations 6–8 of inbred lines. Roz st9 is the third generation, homozygous for six resistance genes against fusarium wilt, late blight, bronze leaf of tomato, verticillium wilt, nematodes, and tomato mosaic virus. All lines exhibit functional male sterility, self-compatibility, and indeterminate growth. Fruits of Roz Son and Roz st9 are pink; st8, green; and st6(lik), st6, and st4, red. The sticky nightshade sample Sn was also obtained from the Timofeev Breeding Station. These plants are characterized by self-incompatibility, determinate growth, and intensely red fruits measuring 2–3 cm in diameter.

Hybridization of tomato and sticky nightshade was conducted from 9 to 11 a.m. The crosses were reciprocal. For the control, the tomato parental lines were self-pollinated. Pollination was performed on emasculated buds: for the tomato, at the lemon-yellow bud stage; for the nightshade, at the white bud stage, one or two days before the flowers opened. Immediately after emasculation, pollination was performed with freshly collected pollen, and the stigma was isolated with cotton wool. A total of 1,165 tomato flowers were pollinated with sticky nightshade, not counting the flowers used for histological analysis. The numbers of pollinations performed by cross combination were: Roz Son – 156, Roz st9 – 298, st8 – 185, st6(lik) – 190, st6 – 132, and st4 – 204. At least five plants of each tomato sample and six plants of nightshade were involved in the hybridization.

Histology. Tomato flowers and developing fruits were fixed in a formaldehyde–alcohol–acetic acid (FAA) solution (Ruzin, 1999). At subsequent steps, a minimum of two samples (flower or developing fruit) were gathered from each plant line up to 15 days after pollination (DAP). They were analyzed either whole or after longitudinal dissection. Individual ovules (at least 10 from each plant line) or ovules attached to the placenta were isolated from samples taken after 15 DAP and used for further study. Samples were dehydrated through a graded ethanol series, cleared in xylene, and embedded in paraffin. Serial sections 10–15 μm thick were obtained with a Microm HM355S rotary microtome (Thermo Fisher Scientific). Sections were stained with 0.1 % Alcian Blue in 3 % acetic acid and 1 % aqueous Safranin (Barykina et al., 2004) or stained with PAS reaction followed by Ehrlich’s hematoxylin staining (Ruzin, 1999). Staining was carried out manually or using a Varistain Gemini ES automated stainer (Thermo Fisher Scientific). Slides were examined under an AxioPlan 2 light microscope (Carl Zeiss) equipped with a differential interference contrast (DIC) module, or with a BX61VS slide scanner (Olympus). Image processing was performed using Adobe Photoshop and ImageJ software.

Embryo rescue. Fruits were selected for cultivation on nutrient media on 16 to 36 days after pollination. Fruit set was counted as the fruits were harvested. The fruits were sterilized in a 2 % sodium hypochlorite solution and then washed three times in sterile distilled water. Enlarged ovules were isolated and transferred to a nutrient medium. We used for ovule culture the nutrient media described by D.J. Harberd (1969) and Ł. Piosik et al. (2019). If the ovule integument had no ruptures and the embryos did not germinate on the solidified nutrient medium, the embryoids were removed from the enlarged ovules after three weeks and cultivated on a medium with the same composition. To regenerate shoots from callus tissue, a solidified Murashige–Skoog (MS) medium containing 30 g/L sucrose, 5 mg/L BAP, and 0.01 mg/L IAA was used. If the ovules germinated or formed organogenic callus with shoots, they were transferred to an MS medium with agar, containing 20 g/L sucrose and 0.01 mg/L each of BAP and IAA. In cases of difficulty in shoot rooting, a MS nutrient medium containing 20 g/L sucrose, 2 mg/L NAA, and 8 g/L agar was used.

Phenotyping of the obtained plants. The st8×Sn (No. 1, 2, and 3) and st6×Sn plants (No. 1, 2, 3, 4, 5, and 6), as well as the parental lines st6, st8, and sticky nightshade Sn, were examined with regard to the morphological characteristics of their shoots, flowers and fruits, in accordance with the DUS testing guidelines of the State Commission of the Russian Federation for Selection Achievements Test and Protection (https://gossortrf.ru/publication/#publication-methodics).

Cytological studies of the chromosome numbers of the obtained plants in mitotic and meiotic cells. Cytological preparations were made from anther and root meristematic cells using the SteamDrop method (Kirov et al., 2014). The prepared slides were examined under an AXIOSKOP 40 microscope (Carl Zeiss) at 1000× magnification using immersion oil. At least 10 cells per sample were examined.

Statistical analysis. Chi-square analysis was used to compare fruit set following the pollination of tomato lines by S. sisymbriifolium. Pearson’s chi-square test was used to assess the significance of differences between genotypes at a significance level of p = 0.05. For pairwise comparisons between genotypes, the Bonferroni correction was applied to adjust the critical significance level. Data analysis and graphical visualization of the results were performed using the Microsoft Excel and R Studio (version 2025.09.2+418) software packages

## Results

**Typical pattern of embryogenesis following self-pollination in S. lycopersicum line Roz st9**

**Fig. 1.**
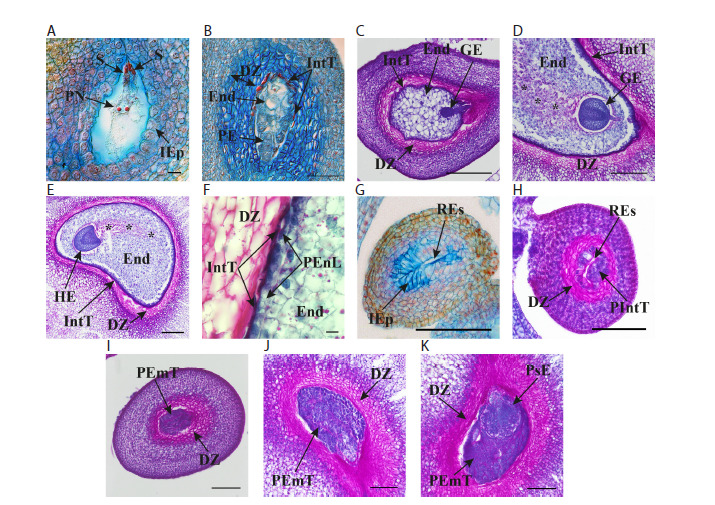
Typical seed development following self-pollination in S. lycopersicum line Roz st9 (A–F) and observed aberrations leading to the formation of seed‑like bodies (G–K). A – structure of the embryo sac and surrounding tissues one day before anthesis (0 DAP); B – endosperm and proembryo in the developing seed, 5 DAP; C – early globular embryo, 15 DAP; D – late globular embryo, 24 DAP; E – developing embryo at the heart stage, 24 DAP; F – endosperm contact zone with surrounding tissues of the developing seed coat, 24 DAP; G – cells of the inner epidermis of the integument grow into the collapsed embryo sac, 5 DAP; H – development of the death zone around the endothelium, 10 DAP; I – pseudoembryonic tissue development, 15 DAP; J – seed-like body filled with pseudoembryonic tissue, 24 DAP; K – pseudoembryo surrounded by pseudoembryonic tissue, 24 DAP. DZ – death zone; End – endosperm; GE – globular embryo; HE – heart-shaped embryo; IEp – inner epidermis of the integument; IntT – integumentary tapetum; PE – proembryo; PEnL – peripheral endosperm cell layer; PN – polar nuclei; PsE – pseudoembryo; S – synergid; PEmT – pseudoembryonic tissue; PIntT – proliferating integumentary tapetum; REs – remnants of the degenerated embryo sac. Asterisks indicate the region of endosperm cells being degraded by the embryo. Scale bars: A, F – 10 μm, B – 50 μm, G, K – 100 μm, C–E – 200 μm.

In order to compare the early stages of embryogenesis, S. lycopersicum line Roz st9 was chosen as a control line. Only mature unitegmic tenuinucellate ovules with completely developed embryo sacs typical of tomatoes were observed in the ovary of control plants one day before anthesis (Fig. 1A). The ovules exhibited a multicellular endosperm and a linear proembryo by day 5 after self-pollination (Fig. 1B). Simultaneously, the integumentary tapetum (IT), also referred to as endothelium, originated from the inner epidermis of the integument, beginning its development on the chalazal pole. The parenchyma cells of the integument adjacent to the IT developed thickened cell walls, and their contents began to degenerate, forming a “death zone” (DZ).

Further seed development was accompanied by an increase in size due to both the proliferation of cells at the periphery of the developing seed coat and the continuous expansion of the embryo and endosperm (Fig. 1C–E). The embryo reached the early globular stage by 10–15 DAP (Fig. 1C). The accumulation of storage sugars began in the endosperm cells, which were tightly attached to the embryo. The outer part of the endosperm was fully covered by a single-layered IT. Spherical embryos with a well-developed suspensor at the late globular stage (Fig. 1D) and embryos at the heart stage (Fig. 1E) were observed in developing seeds at 24 DAP. The endosperm cells adjacent to the IT differed from neighboring cells in having smaller sizes, flattening along the IT, and noticeable vacuolization; thus, they can be considered a separate peripheral layer of endosperm cells (Fig. 1F). The embryos were surrounded by a well-developed endosperm cavity, whereas a number of endosperm cells underwent degradation due to nutrient uptake by the embryo (Fig. 1D, E). At 28 DAP, embryos at the torpedo stage were detected in developing seeds.

**Deviations from the typical embryogenic pattern after self-pollination in S. lycopersicum line Roz st9**

Embryo sac degeneration was the most common deviation in ovule development after self-pollination of tomato lines. Such ovules were characterized by the destruction and shrinkage of the embryo sac by 5 DAP (Fig. 1G). Ovule development ceased when the inner epidermis cells of the integument noticeably elongated and filled the space left by the collapsed embryo sac.

In some cases, the cells of the inner epidermis of the integument did not elongate following embryo sac collapse at 5–10 DAP but instead differentiated into IT and started to proliferate, and this process was accompanied by the formation of DZ around them (Fig. 1H). Such ovules continued to grow and produce seed-like bodies, which were smaller than ordinary seeds. The proliferating IT formed a pseudoembryonic tissue, distinguished by the formation of cells clusters, thicker cell walls at the cluster borders, lack of storage sugars during the subsequent ovule development, intense staining characteristic of IT (Fig. 1I, J), and direct contact with the DZ. One sample collected at 24 DAP contained a body resembling an embryo at the late globular stage at the micropylar side surrounded by pseudoembryonic tissue (Fig. 1K). Since its nature is still unknown, we hereafter refer to such bodies as “pseudoembryos”. It was characterized by a large, broad suspensor that contacted the DZ and by the absence of visible cellular differentiation, including protoderm cells. The cells of the pseudoembryonic tissue were closely attached to the pseudoembryo.

**Pattern of embryogenesis in crosses between S. lycopersicum and S. sisymbriifolium**

**Fig. 2.**
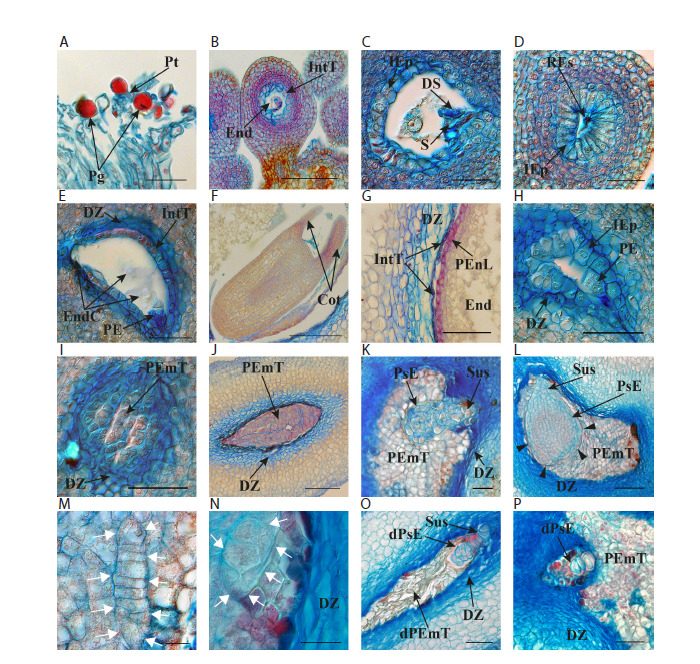
Development of seeds and seed-like bodies after pollination of S. lycopersicum with S. sisymbriifolium pollen. A – germinated pollen grains on the receptive surface of the stigma, 3 DAP; B – early endosperm cell divisions, 3 DAP; C – ovule with degenerated synergid, 3 DAP; D – cells of the inner epidermis of the integument grow into the collapsed embryo sac, 3 DAP; E – proembryo surrounded by cellular endosperm (nuclei out of focus), 15 DAP; F – embryo at the torpedo- shaped stage, 15 DAP; G – endosperm contact zone with surrounding tissues of the developing seed coat, 15 DAP; H – cells of the inner epidermis of the integument grow into the collapsed embryo sac with the presence of the proembryo, 15 DAP; I – seed-like body with pseudoembryonic tissue surrounded by DZ, 15 DAP; J – seed-like body with proliferated pseudoembryonic tissue and developed DZ, 15 DAP; K – pseudoembryo at the globular stage of development, 28 DAP; L – large pseudoembryo at the globular stage of development, 30 DAP; M – cell cluster in the pseudoembryonic tissue, 28 DAP; N – contact zone between pseudoembryonic tissue and DZ, 30 DAP; O – degenerating pseudoembryonic tissue and pseudoembryo, 29 DAP; P – degenerating pseudoembryo with intact pseudoembryonic tissue, 30 DAP. Cot – cotyledons; dPsE – degenerating pseudoembryo; dPEmT – degenerating pseudoembryonic tissue; DS – degenerated synergid; DZ – death zone; End – endosperm; EndC – endosperm cells; IEp – inner epidermis of the integument; IntT – integumentary tapetum; PEmT – pseudoembryonic tissue; PsE – pseudoembryo; REs – remnants of the degenerated embryo sac; S – synergid; Sus – suspensor. White arrows indicate the boundaries of the cell cluster within the pseudoembryonic tissue. Black triangles indicate elongated cells of the pseudoembryonic tissue at the embryo boundary. Scale bars: M, N – 25 μm, C, D – 30 μm, A, E, H, I, K, O, P – 50 μm, B, G, J, L – 100 μm, F – 200 μm.

Germinating pollen grains were observed in all analyzed cross combinations after hybridization between S. lycopersicum and S. sisymbriifolium (Fig. 2A). Only mature ovules with fully developed embryo sacs were observed in all examined ovaries at 1 DAP. Although no growth of pollen tubes into the ovules was observed, at 3 DAP ovules with one or two degenerated synergids were found, and initial divisions of endosperm cells were detected (Fig. 2B, C). The onset of IT differentiation was also noted. In some ovules, the embryo sac degenerated and cells of the inner epidermis of the integument elongated (Fig. 2D). Such ovules were arrested in their development and were present in samples at all subsequent developmental stages.

Samples collected at 10–15 DAP revealed significant variation in the observed stages of embryogenesis depending on the line examined. Seeds following the typical course of embryogenesis for tomatoes were observed in the Roz st9 and Roz Son lines. In developing seeds of the Roz st9 line, a linear proembryo was present together with an endosperm consisting of a small number of cells, which was surrounded by IT and a forming DZ (Fig. 2E). In contrast, in the Roz Son line at 15 DAP, embryos at the torpedo stage surrounded by an endosperm cavity (Fig. 2F), a well-developed endosperm with a differentiated layer of peripheral cells surrounded by IT, and a well-developed DZ were detected (Fig. 2G). In control plants, the corresponding stages of seed development in the Roz st9 and Roz Son lines were observed at 5 and 28 DAP, respectively. In ovary samples of the st6 line at 15 DAP, ovules without discernible changes or with a degenerated synergid were observed.

In all lines examined, most ovules at 15 DAP were arrested in development: their embryo sac degenerated to be replaced by elongated cells of the inner epidermis of the integument. It was noted in some ovules of the Roz st9 line that although the cells of the inner epidermis of the integument began to elongate, cells not belonging to the integument were present on the micropylar side, and the DZ began to differentiate (Fig. 2H). Some seed-like bodies in the Roz st9 line (at an earlier developmental stage) and in the Roz Son line (at a later developmental stage) were occupied by pseudoembryonic tissue, as in the control plants (Fig. 2I, J).In all lines, only two types of seed-like bodies were found in the examined samples at 20–30 DAP: those filled with pseudoembryonic tissue and those containing pseudoembryonic tissue together with an embryo on the micropylar side (Fig. 2K, L). No seeds following the developmental pathway typical of tomato were detected. The pseudoembryonic tissues in both types of seed-like bodies shared some features (Fig. 2M, N): formation of clusters of cells with thicker cell walls at the boundaries of the clusters, absence of storage substances, and direct contact with the DZ without any specialized cells. Some pseudoembryonic tissue cells, most often at the periphery, had granular cytoplasm. They were highly stained with Safranin (Fig. 2L, N).

The drop-shaped pseudoembryo was always located on the micropylar side, and its narrow part, resembling a suspensor, contacted the DZ (Fig. 2K, L). In some pseudoembryos, the suspensor was linear and looked as a single file of cells, whereas in larger pseudoembryos it consisted of several files of cells that widened acropetally, making it impossible to draw a precise boundary between the pseudoembryo and the suspensor (Fig. 2K, L). The outermost cells were relatively flattened only in the largest pseudoembryos (Fig. 2L), whereas in most cases no visible cellular differentiation, including protoderm cells, was observed (Fig. 2K). Some pseudoembryos were covered by a cuticle (Fig. 2K). The adjacent pseudoembryonic tissue cells were always closely appressed to the pseudoembryo. They showed no signs of degradation. In some seed-like bodies, degeneration of both the pseudoembryo and the pseudoembryonic tissue (Fig. 2O) or only of the pseudoembryo itself (Fig. 2P) was observed.

**Fruit set in the hybridization of S. lycopersicum and S. sisymbriifolium, and the cultivation of isolated ovules and embryos**

The average fruit set rate for the beef tomato lines Roz Son and Roz st9 in 2024 was 82.6 ± 2.1 %. For the cherry tomato lines st8, st6(lik), st6 and st4, it was 86.9 ± 6.7 %. No fruit set was observed when nightshade was pollinated with tomato pollen in any of the cross combinations.

**Fig. 3.**
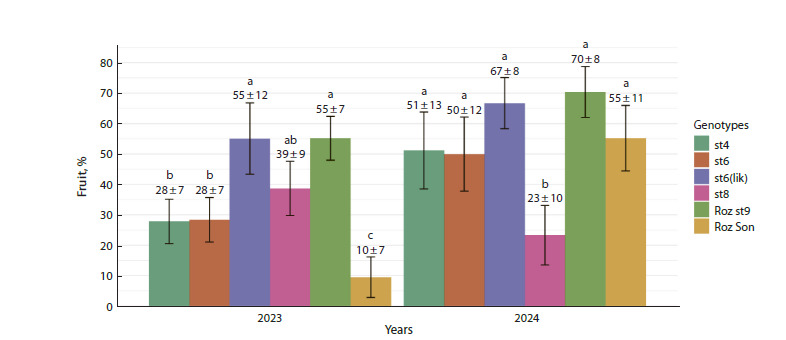
Fruit set in the hybridization of S. lycopersicum and S. sisymbriifolium. Chart bars labeled with the same lowercase letters (a, b, c) do not differ significantly at the 5 % significance level (P ≤ 0.05).

The number of developing ovules and fruit set in crosses between S. lycopersicum and S. sisymbriifolium depended more on the genotype of the maternal plant than on its group membership (Fig. 3). The beef tomato line Roz st9 and the cherry tomato line st6(lik) exhibited high fruit sets. The cherry tomato line st8 exhibited a low fruit set. Fruit set was also influenced by weather conditions of that year; on the average, fruit set was lower in 2023 for all genotypes except st8, though the overall trend was consistent across genotypes.

In 2023, we isolated 123 ovules of the Roz st9×Sn combination, 87 ovules of the Roz Son×Sn combination, 42 ovules of the st6×Sn combination, 72 ovules of the st6(lik)×Sn combination, 45 ovules of the st8×Sn combination, and 88 ovules of the st4×Sn combination. No regenerated plants were obtained from isolated embryos cultured in 2023.

In 2024, we isolated 275 enlarged ovules of the Roz st9×Sn combination, 97 ovules of Roz Son×Sn, 127 ovules of st6×Sn, 304 ovules of st6(lik)×Sn, 117 ovules of st8×Sn, and 178 ovules of st4×Sn. During the cultivation of enlarged ovules on nutrient media, browning or whitening of the ovules with their subsequent death was predominantly observed. An increase in the size of the ovules, rupture of the integument, and germination of the embryo were observed only in the st8×Sn combination in 2 % of the isolated ovules. In the st6×Sn and st6(lik)×Sn combinations, the ovules increased in size without integument rupturing. They were opened manually and the embryos were isolated. Nonorganogenic callus formed from 58 % of the extracted st6×Sn embryos, while organogenic callus formed from 30 %, shoots were obtained from the latter, and the remaining embryos died. In the st6(lik)×Sn combination, organogenic callus formed from only 1 % of the embryos, and plants were obtained from it. As a result, we obtained three plants from the st8×Sn combination, six plants from the st6×Sn combination, two plants from the st6(lik)×Sn combination, and one plant from the st4×Sn combination.

In the in vitro combinations involving beef tomatoes, either embryo death or callus formation was observed. Plant regeneration from the callus was not successful. Callus was obtained from 25 % of the embryos introduced into culture in the Roz Son×Sn combination and from 58 % in the Roz st9×Sn combination

**Morphological traits of the plants obtained**

A detailed analysis of the progeny resulting from the S. lycopersicum × S. sisymbriifolium hybridization and of the parental forms was conducted to identify phenotypic differences from the parental forms. Forty traits were taken into consideration. In what follows, we dwell on the plants derived from the st8×Sn and st6×Sn combinations (Supplementary Material)1.

Supplementary Materials are available in the online version of the paper: https://vavilov.elpub.ru/jour/manager/files/Suppl_Vish_Engl_30_4.pdf


The plants resulting from the S. lycopersicum × S. sisymbriifolium hybridization had an indeterminate growth habit, though the intensity of their growth processes varied. At the time of measurement, the plants obtained from the st8×Sn cross ranged in height from 152 to 186 cm, whereas the st8 line measured 230 cm and the nightshade measured 160 cm. A similar trend was observed in the analysis of the st6×Sn plants: the st6 parent reached a height of 230 cm, whereas the progeny ranged from 150 to 210 cm in height.

The leaf position relative to the stem in the plants obtained from the st6×Sn and st8×Sn hybrid combinations was semierect, as in the tomato parental lines; however, in plants No. 3, 4, and 6 of the st6×Sn combination, a semi-vertical leaf position was observed, which was not characteristic of either parental form. Based on leaf blade characters, both intermediate expressions of traits and the emergence of new traits were identified (Table S1).

**Fig. 4.**
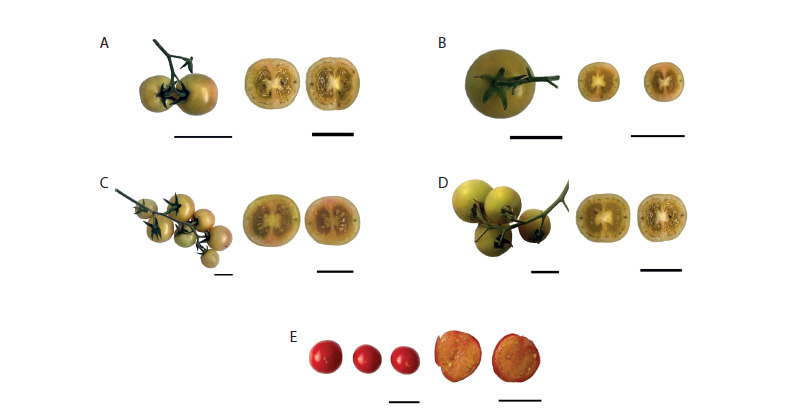
Fruit and pulp coloration in plants obtained from the st8×Sn combination and the parental genotypes: A – st8×Sn No. 1; B – st8×Sn No. 2; C – st8×Sn No. 3; D – tomato line st8; E – S. sisymbriifolium Sn Scale bars: A – 5 cm, B–E – 2.5 cm.

Analysis of the morphological traits of fruits in plants obtained from the st8×Sn hybrid combination revealed intermedi- 1 Supplementary Tables S1 and S2 are available at: https://vavilov.elpub.ru/jour/manager/files/Suppl_Vish_Engl_30_4.pdf ate fruit coloration between the parental forms (Table S1). The st8 tomato had yellow-green mature fruit, while the S. sisymbriifolium was red. All plants obtained from this combination exhibited yellow-orange coloration; the pulp also acquired an uneven yellow-orange tint (Fig. 4), with plants No. 1 and 3 showing more intense coloration than plant No. 2.

Analysis of fruit and pulp coloration in the plants obtained from the st6×Sn combination revealed no segregation: all fruits were orange-red, matching the st6 parental tomato line. However, plant st6×Sn No. 1 exhibited green striping on the fruit prior to ripening, a trait not observed in either parental form. The fruit shape in the longitudinal section demonstrated segregation: plants st6×Sn No. 3, 4, 5, and 6 displayed a heartshaped longitudinal section similar to the st6 line, whereas plant st6×Sn No. 2 was round, like S. sisymbriifolium. The longitudinal section of st6×Sn No. 1 differed from both parents and was oblate.

**Chromosome number analysis in meristems and in microsporogenesis**

**Fig. 5.**
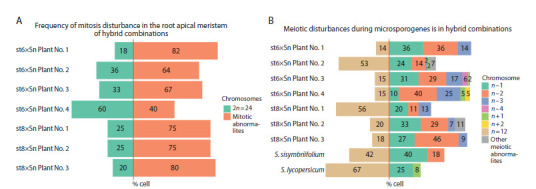
Distribution of cells with the expected chromosome number and cells with altered chromosome numbers (A) in mitosis in meristems and (B) in meiosis during microsporogenesis.

Analysis of the chromosome number in root meristems revealed mixoploidy in all plants obtained from the cross between tomato and S. sisymbriifolium. The chromosome number varied from 16 to 26. The proportion of cells with the expected chromosome number of 2n = 24 varied significantly among the samples (Fig. 5A).

Of the st6×Sn combination, plant No. 4 was the most stable in terms of chromosome number in root meristems. Of the examined cells, 60 % had 24 chromosomes and 40 % had 22. In the other plants studied from the st6×Sn and st8×Sn combinations, the percentage of cells with the expected number of chromosomes was substantially lower, not exceeding 40 % (Fig. 5A). In plant st6×Sn No. 1, 45 % of the examined cells had 26 chromosomes; however, cells with 19, 22, and 25 chromosomes were also noted. In plant st6×Sn No. 2, 36 % of the examined cells had 26 chromosomes, while other cells had 16, 20, or 22 chromosomes. In plant st6×Sn No. 3, 33 % of cells had 26 chromosomes, 25 % had 22, and a few had 20. Chromosome numbers in cells from the st8×Sn plants varied from 20 to 23, and no cells with excess numbers were found.

Analysis of microsporogenesis in the parental tomato forms showed that 33 % of cells had altered chromosome numbers, whereas S. sisymbriifolium showed 58 %. In plants obtained from their hybridization, the proportion of cells with altered chromosome numbers and other meiotic aberrations increased to 86 % (Fig. 5B). The number of chromosomes in the meiotic cells of plants derived from the cross between tomato and S. sisymbriifolium varied broadly, from 8 to 14 chromosomes per cell

**Fig. 6.**
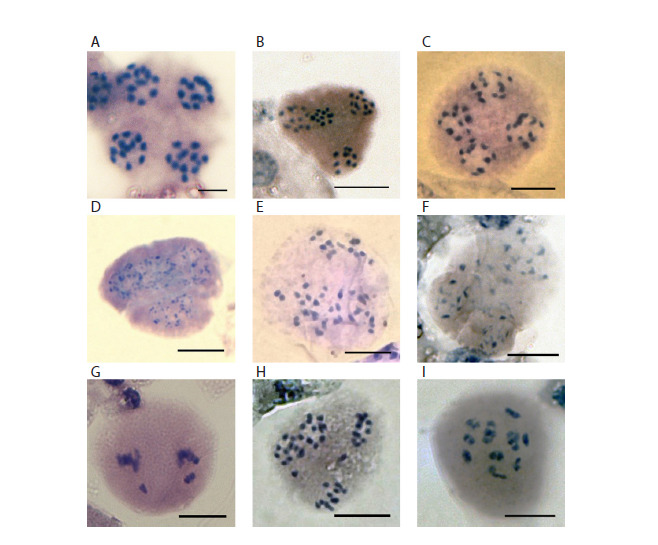
Fragments of cytological preparations with chromosomes of meiotic cells: A – sticky nightshade (telophase II); B – st6×Sn No. 2 (telophase II); C – tomato (telophase II); D – st6×Sn No. 2 (telophase II); E, F – st8×Sn No. 2 (telophase II); G – st8×Sn No. 2 (anaphase I); H – st8×Sn No. 3 (telophase II); I – st6×Sn No. 3 (prophase I). Scale bars 10 μm.

The most frequent division irregularities were those leading to the formation of aneuploid microspores in tetrads with chromosome numbers of n – 1, n –2 and n – 3 (Fig. 5B). Additionally, spontaneous chromosome doubling was observed in plant No. 2 of the st6×Sn combination (Fig. 6D). Division irregularities associated with uneven chromosome distribution during telophase II of meiosis were found in plants No. 1 and 2 of the st8×Sn combination (Fig. 6E, F) and in st6×Sn No. 4. Meiotic irregularities such as nondisjunction or lagging chromosomes during metaphase and telophase II were noted in plants No. 2 and 3 of the st6×Sn cross and plants No. 1–3 of st8×Sn (Fig. 6G, H). We also noted the formation of univalents, trivalents, and bivalents with a single chiasma in st6×Sn No. 2 and 3, and st8×Sn No. 2 (Fig. 6I).

## Discussion

Determining the nature of plants obtained through crossing remains one of the primary challenges in any interspecific hybridization study. Previous reports on S. lycopersicum × S. sisymbriifolium hybrids produced conflicting results. Some researchers classified the progeny as haploids or doubled haploids (Chambonnet, 1996; Bal, Abak, 2003), while others confirmed their status as true interspecific hybrids (Piosik et al., 2019). The plants obtained in this study were phenotypically similar to the tomato parent. This is consistent with the results of U. Bal and K. Abak (2003); however, we hold the view that these plants are of hybrid origin.

Only mature embryo sacs (Fig. 1A) typical of tomatoes (Cooper, 1931) were recognized in both the control line and all examined plants from lines pollinated with S. sisymbriifolium pollen one day before anthesis. Accordingly, all aspects of ovule development observed in this study are linked to fertilization or post‑zygotic stages of development. In the analyzed S. lycopersicum lines, four variants of ovule development were observed following either self-pollination or hybridization with S. sisymbriifolium. During the typical developmental course (Fig. 1B–F, 2A–C, 2E–G), normal tomato seeds formed, characterized by a well‑developed embryo surrounded by a cellular endosperm, IT, and DZ. In addition, three alternative developmental types were detected: (i) developmental arrest (Fig. 1G; 2D); (ii) formation of pseudoembryonic tissue (Fig. 1H–J; 2G, I); and (iii) formation of pseudoembryonic tissue with a pseudoembryo (Fig. 1K; 2K–P). The last two patterns resulted in the production of seed‑like bodies.

The most prevalent type of ovule development in all examined plants was developmental arrest caused by the collapse of the embryo sac and the filling of the resulting gap by elongated inner epidermal cells of the integument (Fig. 1G; 2D) without IT differentiation. This variant of ovule development has been described by several authors, and the origin of the observed elongated cells from the inner epidermis cells of

the integument is unquestionable (Palamarchuk et al., 1975; Chaban et al., 2020). However, I. Chaban et al. (2020) reported that ovules with this type of development undergo further development, which was not observed in our study.

A third alternative outcome of the interspecific hybridization (Fig. 2K–P) rather than of self‑pollination (Fig. 1K) proved to be more typical. A pseudoembryo surrounded by pseudoembryonic tissue developed inside seed‑like bodies, a pattern not reported in earlier studies (Chambonnet, 1996; Bal, Abak, 2003; Piosik et al., 2019). In these cases, all observed pseudoembryos reached the globular or late‑globular stage (Fig. 1K; 2K, L). Numerous studies on Solanaceae hybrids have shown that seed development often stops at the globular stage, which marks a critical step in overcoming the post‑zygotic interspecific barrier to crossability (Baek et al., 2016; Roth et al., 2018; Piosik et al., 2019). Scientists, however, seldom discuss the nature of the tissues surrounding the embryo and often overlook the possibility of pseudoembryonic tissue formation in tomato. Hybrid seeds are usually described as either developing a normal endosperm or having degenerated endosperm accompanied by the formation of a multilayered IT (Baek et al., 2016; Roth et al., 2018), which does not resemble the pseudoembryonic tissue observed in this study.Particular consideration should be given to the characteristics of the pseudoembryos surrounded by pseudoembryonic tissue. Similar abnormalities have been reported in several angiosperms with unitegmic ovules, where adventive embryos may arise from IT (Kapil, Tiwari, 1978). In our material, however, each pseudoembryo developed strictly at the micropylar pole, which contrasts with adventive embryos originating from IT in random sites. Evidence supporting hybrid origin comes from ovules at early stages after pollination, where cells presumably corresponding to an embryo were found at the same position, the micropylar pole, despite the beginning of IT proliferation (Fig. 2H). These observations suggest that pseudoembryonic tissue forms from IT, while the embryo develops at the micropylar pole. As we did not observe endosperm degeneration in ovules containing a pseudoembryo, it seems unlikely that the seed‑like bodies resulted from replacement of degenerated endosperm with pseudoembryonic tissue. Taken together, our findings indicate that the pseudoembryos formed in this study may be of hybrid origin.

In this research, we isolated ovules on nutrient medium. Consequently, the in vitro material consisted of normally developing seeds along with seed-like bodies, either parthenocarpic or possessing embryos. During the cultivation of isolated ovules, relatively rapid embryo and plantlet growth on the nutrient medium was observed only in the st8×Sn combination. The plantlets rapidly grew upon transplanting into soil, which is consistent with the observations of U. Bal and K. Abak (2003). In the combinations st6×Sn, st4×Sn, and st6(lik)×Sn, plant regeneration occurred through the formation of organogenic callus from the isolated embryoids. These plantlets developed slowly, and the majority of them failed to root. A similar growth trend was noted by Ł. Piosik et al. (2019)

With regard to morphological traits, all obtained plants resembled S. lycopersicum, consistent with the results of U. Bal and K. Abak (2003) and D. Chambonnet (1996). This is notable because interspecific tomato hybrids often exhibit phenotypes with prominent features of the paternal component (Gavrilenko et al., 2001; Piosik et al., 2019). However, detailed analysis of the phenotypic characteristics of the studied plants revealed certain traits of S. sisymbriifolium, along with the emergence of traits absent from either parental form. We rule out the possibility of phenotypic segregation in the progeny of the parental tomato line, as we employed genetically stable multigenerational inbred lines

Cytogenetic analysis of root meristems revealed mixoploidy, with chromosome numbers ranging from 16 to 26. U. Bal and K. Abak (2003) also reported the occurrence of mixoploidy (2n = 24–26) in the meristems of plants derived from S. lycopersicum × S. sisymbriifolium hybridization; however, they interpreted this phenomenon as a sign of chromosome doubling in haploid plants. In contrast to the results of U. Bal and K. Abak (2003), a significant proportion of cells in our study contained fewer than 24 chromosomes. Notably, plants from the st8×Sn combination exhibited no cells with an increased chromosome count. This reduction is likely attributable to the elimination of S. sisymbriifolium chromosomes. Mixoploidy in meristems can serve as both a marker of hybrid origin and a response to plant regeneration through callus culture. However, the st8×Sn plants were obtained through direct morphogenesis without a callus stage, which further supports their hybrid origin. Furthermore, E.W. Lindstrom and K. Koos (1931) obtained a doubled haploid from the callus of a haploid tomato; notably, its microsporogenesis was characterized as regular, producing microspores with a consistent count of n =12 chromosomes.

Researchers (Chambonnet, 1996; Bal, Abak, 2003; Piosik et al., 2019) who experimented with the hybridization of S. lycopersicum and S. sisymbriifolium did not study microsporogenesis in the obtained plants. T. Gavrilenko et al. (2001) obtained a somatic hybrid of S. lycopersicum and S. etuberosum and noted the presence of univalents in meiotic cells, as well as lagging chromosomes during anaphase, meiosis telophase I, and prophase II. Our study also revealed numerous meiotic irregularities in the obtained plants, manifesting themselves in chromosome lagging, uneven distribution of chromosomes between daughter cells, and the formation of univalents, trivalents, and bivalents with a single chiasma. Meanwhile, the S. lycopersicum and S. sisymbriifolium lines used in our study had aneuploid cells (n – 1), which may be attributed to high temperatures (33–37 °C) during plant cultivation (Schindfessel et al., 2023).

On the grounds of the multitude of processes studied that follow the pollination of S. lycopersicum with S. sisymbriifolium pollen, the production of plants in embryoculture and the differences in phenotypes between the parental tomato lines used and the obtained plants, and their cytogenetic features, we consider the obtained plants to be interspecific hybrids. Further research is required to confirm the hybrid origin of the obtained plants, including the development of specific molecular markers or the optimization of the GISH (genomic in situ hybridization) protocol, which is beyond the scope of this study. However, this does not diminish the importance of our results, which help us understand what happens during the hybridization of these species. They also show that there is a chance that valuable traits from S. sisymbriifolium can be added to the resulting plants, as we have shown that they can produce fruit.

## Conclusion

In crosses between S. lycopersicum and S. sisymbriifolium, two alternative developmental types leading to the formation of seed‑like bodies were found in developing fruits, in addition to normally developing seeds and ovules arrested during early development. Some of these seed‑like bodies represented parthenocarpic seeds filled with pseudoembryonic tissue formed as a result of proliferation of the integumentary tapetum. Certain seed‑like bodies containing pseudoembryonic tissue also enclosed an embryo arrested at the globular stage. Its position at the micropylar pole and the absence of evidence for an adventive origin suggest that the embryo may be of hybrid nature. The developmental arrest of embryos and the formation of pseudoembryonic tissue in place of endosperm provide further evidence for postzygotic barriers that hinder the production of interspecific hybrids between S. lycopersicum and S. sisymbriifolium

We used the method of isolating ovules and embryos from them to hybridize cherry tomato lines and S. sisymbriifolium and obtained 12 viable plants. While all of the obtained plants had tomato-like phenotypes, they demonstrated high chromosomal instability in their meristematic cells. Additionally, we studied the microsporogenesis process for the first time in S. lycopersicum and S. sisymbriifolium crosses and discovered a significant number of meiosis I and II abnormalities, such as uneven chromosome distribution between daughter cells, lagging chromosomes, and the formation of univalents and trivalents. This finding supports the hypothesis that the obtained samples are hybrids.

The obtained results add to our understanding of the processes occurring during distant hybridization and open up the possibility of using S. sisymbriifolium as a source of valuable traits in tomato breeding.

## Conflict of interest

The authors declare no conflict of interest.
